# Nurse-led digital health program for home blood pressure monitoring in stroke patients: protocol for a pooled analysis of randomized controlled trials

**DOI:** 10.3389/fpubh.2024.1378144

**Published:** 2024-07-22

**Authors:** Wei Zhang, Zubing Mei, Zaibang Feng, Bin Li

**Affiliations:** ^1^Department of Neurology, Xinxiang Central Hospital, The Fourth Affiliated Hospital of Xinxiang Medical College, Xinxiang, Henan, China; ^2^Department of Anorectal Surgery, Shuguang Hospital, Shanghai University of Traditional Chinese Medicine, Shanghai, China; ^3^Anorectal Disease Institute of Shuguang Hospital, Shanghai, China; ^4^Department of Rehabilitation Medicine and Physiotherapy, Changhai Hospital, Naval Military Medical University, Shanghai, China; ^5^Department of Neurosurgery, Tongji Hospital, Tongji University School of Medicine, Shanghai, China

**Keywords:** nurse-led digital health, home blood pressure monitoring, stroke, randomized controlled trials (RCTs), telehealth interventions

## Abstract

**Background:**

Hypertension is a major risk factor for stroke recurrence in stroke patients. Home blood pressure monitoring, facilitated by digital health technologies and led by nurses, may improve blood pressure control in this high-risk population. However, the evidence is not yet conclusive. This study protocol outlines a pooled analysis of the current literatures to evaluate the effectiveness of nurse-led digital health programs for home blood pressure monitoring in stroke patients.

**Methods and analysis:**

We will conduct a comprehensive search of some major electronic databases (e.g., PubMed, EMBASE, Cochrane Library, and CINAHL) and trial registries for randomized controlled trials evaluating nurse-led digital health programs for home blood pressure monitoring in stroke patients. Two reviewers will independently screen titles and abstracts, review full-text articles, extract data, and assess risk of bias using the revised Cochrane risk-of-bias tool for randomized trials (RoB 2.0). The primary outcome measures will be changes in both systolic and diastolic blood pressure from baseline to the end of the intervention period. Secondary outcomes include adherence to the program, patient satisfaction, and stroke recurrence. Data will be pooled and analyzed using meta-analysis techniques, if appropriate.

**Discussion:**

This study will provide comprehensive evidence on the effectiveness of nurse-led digital health programs for home blood pressure monitoring in stroke patients. The findings could have substantial implications for clinical practice and health policy, potentially informing the development of guidelines and policies related to hypertension management and stroke prevention.

**Conclusion:**

By pooling the results of randomized controlled trials, this study will offer a robust evidence base to inform clinical practice and health policy in the context of stroke patients. Despite potential limitations such as heterogeneity among studies and risk of publication bias, the rigorous methodology and comprehensive approach to data synthesis will ensure the reliability and validity of the findings. The results will be disseminated through a peer-reviewed publication and potentially at relevant conferences.

**Registration DOI:**

https://doi.org/10.17605/OSF.IO/59XQA.

## Introduction

Stroke is a global health burden, ranking as a leading cause of death and disability worldwide (1, 2). Among the various modifiable risk factors, hypertension stands as an important contributor to stroke incidence, particularly in stroke patients, emphasizing the need for effective blood pressure management strategies (3, 4). Home blood pressure monitoring (HBPM) has emerged as a crucial component of hypertension management. The benefits of HBPM extend beyond the mere measurement of blood pressure. By enabling frequent and regular monitoring, HBPM captures the daily fluctuations in blood pressure, providing a more comprehensive profile than isolated clinic measurements. This aspect is particularly relevant in the context of ‘white-coat’ and ‘masked’ hypertension, where clinic measurements can lead to misclassification (5, 6). Beyond diagnosis, HBPM also fosters patient engagement in their care, which has been shown to improve adherence to antihypertensive treatment and lifestyle changes, thereby enhancing blood pressure control (7, 8). Nevertheless, the effectiveness of HBPM is contingent upon several factors, including the accuracy of measurements, patient adherence to the monitoring schedule, and the interpretation and response to the readings. These considerations underscore the need for strategies to optimize the use of HBPM in stroke patients (9).

Digital health programs, built on advancements in telecommunication and information technology, offer a novel approach to chronic disease management (10, 11). These programs facilitate real-time monitoring, providing immediate feedback based on the monitored parameters. This feature is particularly beneficial for blood pressure control in stroke patients, where timely intervention in response to elevated readings can prevent hypertensive emergencies and end-organ damage (12–14). Furthermore, digital health programs can break the geographical and temporal barriers to healthcare access, offering a convenient and flexible solution for stroke patients who may have mobility limitations or live in remote areas (15, 16).

Nurses, as the largest group of healthcare professionals, have a crucial role in the management of hypertension (17, 18). Their roles extend beyond patient care to include patient education, medication management, and follow-up, which are essential components of hypertension management, particularly for stroke patients (19–21). From a theoretical perspective, nursing as a discipline is centered on holistic patient care and the support of individuals through health transitions. This focus is particularly relevant in the context of chronic illnesses like stroke, where patients often face significant lifestyle adjustments and ongoing health challenges. Nurses are uniquely positioned to guide patients through these transitions, providing continuous support and fostering self-management skills that are critical for long-term health maintenance. Evidence from numerous studies and systematic reviews supports the efficacy of nurse-led interventions in improving blood pressure control (18, 19, 22). A recent systematic review by Kappes et al. highlighted the effectiveness of nurse-led telehealth interventions in reducing hypertension, underscoring the potential for such strategies to improve patient outcomes (23). In the digital health landscape, nurses can take the helm in implementing the program, providing education and support to patients, interpreting and responding to the HBPM readings, and liaising with physicians for medication adjustments, if necessary (24, 25). This integration of digital health tools with traditional nursing care not only enhances the capacity of nurses to manage hypertension effectively but also supports patients in adapting to their new health realities, thereby improving overall health outcomes.

Despite the substantial evidence supporting nurse-led interventions and the growing interest in digital health technologies, there is a notable gap in the literature regarding the combined effect of these two approaches in managing blood pressure among stroke patients. The present study aims to bridge this gap by investigating the effects of a nurse-led digital health program on HBPM in stroke patients through a comprehensive literature review and pooled analysis of published randomized controlled trials (RCTs).

This study is the first to synthesize evidence from high-quality RCTs to evaluate the combined impact of nurse-led care and digital health interventions on blood pressure control in stroke patients. By doing so, it adds to the existing literature by providing robust evidence on the efficacy of integrating nurse-led interventions with digital health technologies, highlighting the potential for enhanced patient outcomes through such innovative approaches.

The primary objective is to determine whether this intervention can lead to improved blood pressure control compared to standard care. Secondary objectives include assessing the impact of the intervention on patient adherence to the HBPM schedule, patient satisfaction with the program, and the incidence of recurrent strokes and other cardiovascular events. These findings could have significant implications for clinical practice and health policy, informing the development of guidelines and strategies for hypertension management and stroke prevention that leverage the strengths of both nurse-led care and digital health technologies.

## Methods

### Study registration

The study will be conducted following the methodological standards set forth in the Cochrane Handbook for Systematic Reviews of Interventions (26). This will guide our review process, data collection, and statistical analysis, ensuring a rigorous and unbiased approach to evidence synthesis. In alignment with the PRISMA-P (Preferred Reporting Items for Systematic review and Meta-Analysis Protocols) guidelines (Supplementary Table 1), this protocol will outline a detailed methodology for a pooled analysis of RCTs examining the effects of nurse-led digital health programs on home blood pressure monitoring in stroke patients. PRISMA-P provides a framework for the systematic planning and reporting of meta-analysis protocols, ensuring comprehensive documentation of our research intentions and methods (27).

This study’s protocol has been proactively registered with the Open Science Framework (OSF) to ensure transparency and reproducibility of the research process. The registration details the objectives, inclusion and exclusion criteria, search strategy, outcome measures, and methods of analysis for the proposed pooled analysis, focusing on stroke patients. The registration DOI number for this study is 10.17605/OSF.IO/59XQA. The full details of the study can be accessed on the PROSPERO website^1^ using the registration number.

### Study design

The study will employ a pooled analysis of RCTs based on both the study level data and level of individual participant data (IPD) whatever data are available, which are considered the optimal approach to minimize confounding factors and bias. Specifically, this analysis will focus on RCTs that have investigated the effects of nurse-led digital health programs on home blood pressure monitoring in patients with a history of stroke. The pooling of data in this context will not only be at the individual participant data (IPD) level but also at the aggregate data level. This means that summary statistics from each study, along with the raw patient data, will be combined to enhance statistical power and improve the precision of the estimated effects of the intervention. This approach will allow us to integrate findings from multiple studies, providing a more robust and generalizable understanding of the nurse-led interventions’ impact on blood pressure control among stroke survivors. Each selected RCT will be carefully assessed for compatibility in terms of intervention components, patient populations, and outcomes measured to ensure validity in the synthesis of the data.

### Data sources

The search for studies to include in the pooled analysis will be a systematic process involving multiple electronic databases. MEDLINE (via PubMed), EMBASE, Cochrane Central Register of Controlled Trials (CENTRAL), and CINAHL will be searched as they cover a broad range of biomedical literature, including nursing and allied health literature. A health sciences librarian will be consulted to develop a robust search strategy, which will include MeSH terms and free-text terms related to stroke, hypertension, home blood pressure monitoring, digital health, and nurse-led interventions (Table 1). Additionally, the reference lists of the included studies and relevant systematic reviews will be examined for potential studies. Clinical trial registries like ClinicalTrials.gov and WHO International Clinical Trials Registry Platform will also be searched for ongoing and completed trials.

**Table 1:** Search strategy for Pubmed database.

Pubmed search terms	
	Stroke terms:
1	Stroke[Mesh]
2	“Cerebrovascular Disorders”[Mesh]
3	“Brain Injury, Chronic”[Mesh]
4	“Hemiplegia”[Mesh]
5	“Paresis”[Mesh]
6	“Gait Disorders, Neurologic”[Mesh]
7	(cerebral or cerebellar or brain* or vertebrobasilar) [Title/Abstract]
8	(infarct* or isch?emi* or thrombo* or emboli* or apoplexy) [Title/Abstract]
9	7 AND 8
10	(cerebral or brain or subarachnoid) [Title/Abstract]
11	(haemorrhage or hemorrhage or haematoma or hematoma or bleed*)[Title/Abstract]
12	10 AND 11
13	(hempar* or hemipleg* or brain injur*) [Title/Abstract]
14	1 OR 2 OR 3 OR 4 OR 5 OR 6 OR 9 OR 12 OR 13
	Blood Pressure/Diabetes terms:
15	“Blood Pressure”[Mesh] or “Diabetes Mellitus”[Mesh]
16	“Hypertension”[Mesh]
17	“Antihypertensive Agents”[Mesh]
18	(hypertens* OR blood pressure)[Title/Abstract]
19	((control* OR elevat* OR high* OR lower* OR rais*) AND (blood pressure OR bp))[Title/Abstract]
20	15-19/OR
	Virtual Healthcare terms:
21	“Telemedicine”[Mesh]
22	(technolog* OR telerehabilitation OR e-rehabilitation OR telecare OR telemedicine OR telehealth OR telecommunication OR telemonitor* OR telenursing OR tele-nursing OR ehealth OR e-health OR mhealth OR m-health OR telecoaching OR gerontechnology OR videoconferenc* OR teleconferenc* OR internet OR computer OR mobile OR phone OR smartphone OR telephone OR tablet OR email OR e-mail OR SMS OR apps OR applications OR “social media” OR wireless OR virtual OR remote OR digital OR distant)[Title/Abstract]
23	21 OR 22
	Nurse-led terms:
24	“Nurses”[Mesh]
25	“Nursing”[Mesh]
26	nurs* OR nurse-led OR nurse led OR nurse-based OR nurse based OR interdisciplin* OR multi-disciplin* OR multidisciplin*
27	24-26/OR
	Study design terms:
28	randomized controlled trial[Publication Type] OR controlled clinical trial[Publication Type]
29	(Randomized or randomised or placebo or drug therapy or randomly or trial or groups) [Title/Abstract]
30	28 OR 29
	Final search results: Combining Stroke and Blood Pressure/Diabetes and Virtual Healthcare and Nurse-lead and Study design:
31	14 and 20 and 23 and 27 and 30

### Eligibility criteria

The selection of studies for the pooled analysis will be based on the PICOS (Population, Intervention, Comparison, Outcome, Study design) framework. The inclusion criteria are as follows:

Population: Adult patients (aged 18 years or older) with a history of stroke. This includes patients who have experienced any type of stroke, including ischemic stroke, hemorrhagic stroke, and transient ischemic attack (TIA). The inclusion of various stroke types will allow for a comprehensive evaluation of the nurse-led digital health program’s effectiveness across different stroke populations.

Intervention: The intervention under review is a nurse-led digital health program for home blood pressure monitoring (HBPM). This program is designed to empower patients with a history of stroke to manage their blood pressure at home, with nurses playing a key role in the process. The digital health program includes a home blood pressure monitoring device, a digital platform (such as a mobile app or a web-based system), and a team of nurses who provide remote support and guidance to the patients.

Patients are trained to use the home blood pressure monitoring device and the digital platform. They are instructed to measure their blood pressure at regular intervals, typically twice a day – once in the morning and once in the evening – and to record the readings on the digital platform. The platform allows for real-time transmission of the readings to the nurses, who monitor the data and provide feedback to the patients. The feedback may include advice on lifestyle modifications, medication adherence, and when to seek medical attention. The nurses also provide emotional support and education to the patients, helping them to understand their condition and the importance of blood pressure control (12).

Comparison: Usual care or any other non-nurse-led interventions for home blood pressure monitoring.

Outcome: The primary outcome measures for this study are the changes in systolic and diastolic blood pressure levels from baseline to the end of the intervention period. Blood pressure, comprising both systolic and diastolic components, will be measured using standard procedures, with readings recorded on the digital platform forming the basis for our analysis. A significant reduction in both systolic and diastolic blood pressures will be regarded as evidence supporting the effectiveness of the nurse-led digital health program in stroke patients (28).

Secondary outcomes include adherence to the program, patient satisfaction, and stroke recurrence. In assessing adherence to the nurse-led digital health program, we will employ a rigorous and consistent approach across all included studies. Where possible, adherence will be measured using objective indicators, such as the number of days the patient engaged with the digital platform, frequency of blood pressure measurements taken, and consistency in uploading these measurements as recorded by the system. For studies where such objective data are unavailable, we will consider self-reported adherence measures, acknowledging their inherent limitations. To ensure standardization, we will adopt a pre-defined threshold for adherence, such as completing at least 80% of the recommended monitoring schedule. We recognize the heterogeneity in how adherence is defined and measured across studies; thus, sensitivity analyses will be performed to evaluate the robustness of our findings to different definitions of adherence. Patient satisfaction will be evaluated using validated questionnaires, such as the Patient Satisfaction Questionnaire Short Form (PSQ-18) (29). Stroke recurrence will be determined by reviewing the patients’ medical records and/or self-reports.

Study design: RCTs.

Exclusion criteria include non-randomized studies, studies not reporting our outcomes of interest, and studies where the necessary data for the pooled analysis cannot be obtained. Studies with unclear definition of the nurse’s role in the digital health program or where the intervention is not primarily nurse-led will also be excluded.

### Study selection

The process of study selection for the pooled analysis will involve several stages, starting with an initial screening of titles and abstracts. Two independent reviewers will conduct this screening to identify potentially relevant studies based on the PICOS criteria outlined in the eligibility criteria. Any disagreements between the reviewers will be resolved through discussion or by consulting a third reviewer.

The full texts of the potentially relevant studies will then be obtained and assessed for eligibility. The same two reviewers will independently conduct this assessment, using a standardized form to ensure consistency. The form will include items related to the study population, intervention, comparison, outcomes, and study design. Studies that meet all the eligibility criteria will be included in the pooled analysis. A flow diagram following the PRISMA guidelines will be used to document the study selection process (25).

### Data extraction

In the data extraction phase, we will meticulously employ a standardized form that has been pilot-tested on select studies to gather all pertinent data points. This task will be carried out independently by two reviewers to reduce the likelihood of errors or bias. All discrepancies encountered during the extraction will be resolved through thorough discussion and mutual agreement, or, if needed, consultation with a third reviewer. We will not use specific software during the data extraction process.

The data items to be extracted will include study characteristics (e.g., authors, year of publication, country), participant characteristics (e.g., age, gender, medical history), details of the intervention and comparison (e.g., components of the nurse-led digital health program, frequency and duration of blood pressure measurements), outcomes (e.g., blood pressure levels, adherence, patient satisfaction, stroke recurrence), and study results (e.g., effect estimates, confidence intervals).

### Risk of bias assessment

The risk of bias in the individual studies will be assessed using the Cochrane Collaboration’s tool for assessing risk of bias in randomized trials (RoB 2.0) (30). This tool evaluates the risk of bias in five domains: randomization process, deviations from intended interventions, missing outcome data, measurement of the outcome, and selection of the reported result. Each domain is rated as low risk, some concerns, or high risk of bias. The overall risk of bias for each study is then determined based on the domain ratings.

Two reviewers will independently conduct the risk of bias assessment, and any disagreements will be resolved through discussion or by consulting a third reviewer. The results of the risk of bias assessment will be presented in a ‘Risk of bias’ summary figure, following the Cochrane Handbook guidelines (26).

### Grading of evidence

The grading of evidence in this protocol will be systematically conducted following the Grading of Recommendations, Assessment, Development and Evaluations (GRADE) approach. This method is recognized for its clear and transparent process of rating the quality of evidence and strength of recommendations (31). For each outcome, the quality of evidence will be assessed across the domains of risk of bias, inconsistency, indirectness, imprecision, and publication bias. Evidence quality will be categorized into four levels: high, moderate, low, or very low. These ratings will reflect our confidence in the effect estimates and the likelihood that further research could impact our confidence in the estimate of effect or change the estimate. The GRADE approach will also allow us to consider the balance between desirable and undesirable effects, the values and preferences of patients, and resource implications, which are particularly relevant in the context of nurse-led digital health interventions for stroke patients. The results of the GRADE assessment will be presented in a ‘Summary of Findings’ table, which will provide a concise and user-friendly overview of the key information that is critical for decision-making (32).

### Statistical analysis

The data will be pooled and analyzed using meta-analysis techniques, if appropriate. The choice of a fixed-effect or random-effects model will depend on the level of heterogeneity among the included studies, which will be assessed using the *I*^2^ statistic (33). If substantial heterogeneity is detected (*I*^2^ > 50%), a random-effects model will be used, and potential sources of heterogeneity will be investigated through subgroup analyses or meta-regression. Subgroup analyses will be performed based on predefined clinical or methodological characteristics. These may include, for example, the intensity of the intervention, duration of follow-up, or baseline blood pressure levels. If there is substantial heterogeneity that cannot be explained by subgroup analyses, we will conduct meta-regression analyses to investigate the impact of continuous variables on the effect size.

To assess for publication bias, which may skew the results if studies with non-significant results are less likely to be published, we will visually inspect funnel plots for asymmetry for each meta-analyzed outcome when we have a sufficient number of studies (typically 10 or more). Additionally, we will employ more quantitative methods such as Egger’s test to statistically assess the funnel plot asymmetry (34). Sensitivity analyses will be conducted to evaluate the robustness of our findings by systematically excluding certain studies that do not meet specific quality criteria or by removing each study one at a time, we can assess the impact of individual studies on the overall meta-analysis results. The trim and fill method will be used in the presence of funnel plot asymmetry suggestive of publication bias. This non-parametric method estimates the number and outcomes of potentially missing studies and adjusts the meta-analysis to account for the hypothesized missing data, providing a more accurate estimate of the effect size (35).

For each outcome, the effect estimates (e.g., mean differences for continuous outcomes, risk ratios for dichotomous outcomes) and their 95% confidence intervals will be calculated. Forest plots will be used to graphically present the results of the meta-analysis. If meta-analysis is not appropriate due to high heterogeneity or other reasons, a narrative synthesis of the results will be conducted.

The statistical analyses will be performed using Stata 12.0 software (Stata Corp LP, College Station, TX, USA).

### Amendments

The protocol governing this pooled analysis is designed to be a living document, subject to modifications and refinements as required throughout the course of the review. Adjustments to the protocol will be judiciously considered and implemented to ensure the review remains current and methodologically sound.

## Discussion

### Expected results

The expected results of this study are to provide comprehensive evidence on the effectiveness of nurse-led digital health programs for home blood pressure monitoring in patients with a history of stroke. Given the increasing use of digital health technologies and the pivotal role of nurses in patient care, it is anticipated that such programs could lead to significant improvements in blood pressure control (36). Previous systematic review using six controlled clinical trials and one quasi-experimental study has suggested that nurse-led interventions can reduce blood pressure in hypertensive patients and patient adherence to treatment (23). Naqvi et al. highlighted the positive impact of remote blood pressure monitoring and telehealth on patient-reported outcomes in stroke care (14). Moreover, digital health technologies have been shown to enhance self-monitoring and self-management of blood pressure for patients with chronic disease including stroke survivors and hypertension, as evidenced by recent studies (37–40). Therefore, combining these two approaches could potentially yield even better results. However, the extent of these benefits and their impact on patient outcomes such as stroke recurrence and complication management are yet to be fully elucidated.

Previous research has suggested that nurse-led interventions can improve blood pressure management and patient adherence to treatment (19). Moreover, digital health technologies have been shown to enhance self-monitoring and self-management of blood pressure (12). Therefore, combining these two approaches could potentially yield even better results. However, the extent of these benefits and their impact on patient outcomes such as stroke recurrence and complication management are yet to be fully elucidated.

### Potential implications

The successful validation of our nurse-led digital health program for home blood pressure monitoring in stroke survivors stands to yield multifaceted implications with profound impacts. Foremost, it introduces a clinically efficacious strategy for hypertension management, alleviating pressures on overburdened healthcare systems by offering a scalable, remote care option (41). This not only streamlines resource allocation but also strengthens the resilience of healthcare infrastructures, especially in managing chronic conditions in the community setting.

Secondly, by fostering patient autonomy, our program is poised to transform patient engagement, invigorating adherence to therapeutic regimens and motivating positive lifestyle adjustments. These behavioral changes are cornerstones of effective blood pressure control, pivotal for both primary and secondary stroke prevention, as well as mitigating complications associated with hypertension (42). By nurturing a culture of self-management, we empower patients to become active partners in their own health journey.

Lastly, the program’s efficacy may catalyze a paradigm shift in healthcare policy and practice guidelines. It underscores the value of integrating digital health innovations with the personalized, supportive care provided by nurses, advocating for their expanded roles in the digital health era. This could prompt the formulation of evidence-based policies that systematically incorporate nurse-led digital interventions into standard care pathways, thereby aligning healthcare strategies with technological advancements and the evolving needs of patients.

Our findings may drive investments toward nursing empowerment and the wider adoption of digital health tools, promising a future of more accessible, patient-centered, and efficient healthcare delivery. The study’s results could support policy development promoting digital health integration into nursing practice, enhancing patient monitoring and management for chronic conditions like hypertension in stroke patients. Success in these programs may necessitate revisions in nursing education to include digital health competencies and telehealth, ensuring nurses are adequately trained. Policies should address resource allocation for digital tools, training programs, and infrastructure, and establish standardized guidelines and protocols to ensure consistency and quality in care delivery.

### Strengths

One of the main strengths of this pooled analysis is the rigorous and systematic approach to study selection, data extraction, and risk of bias assessment, which will enhance the reliability and validity of the findings. Moreover, by including only randomized controlled trials, the highest level of evidence will be obtained, strengthening the conclusions drawn from the analysis. Another strength is the focus on both primary and secondary outcomes, which will provide a comprehensive evaluation of the intervention’s effectiveness. Not only will this include the impact on blood pressure levels, but also adherence to the program, patient satisfaction, and stroke recurrence, providing a holistic view of the intervention’s potential benefits.

### Limitations

Despite these strengths, potential limitations should be acknowledged. Firstly, there may be a high level of heterogeneity among the included studies due to differences in the design and implementation of the nurse-led digital health programs, patient populations, and outcome measures. This could complicate the interpretation of the pooled results. Secondly, the risk of publication bias, where studies with positive results are more likely to be published, cannot be overlooked. Although efforts will be made to minimize this bias by searching for unpublished studies in clinical trial registries, it may still impact the findings. Thirdly, our search strategy is limited by the databases we have selected. Some relevant studies may not be indexed in these databases, leading to incomplete retrieval of eligible studies. Fourthly, the specificity of our search strategy, despite being comprehensive, may result in some relevant studies being missed due to variations in terminology and indexing. Fifthly, reporting bias may affect the quality and completeness of the data we extract, impacting the synthesis process. Sixthly, time lag bias could occur, where there may be a delay between the completion of a study and its publication, resulting in the potential exclusion of recent studies that have not yet been published. Lastly, the quality of the included studies may vary, and studies with a high risk of bias could potentially skew the results. However, to enhance the robustness and reliability of our study findings, we will conduct thorough risk of bias assessments, sensitivity analyses, and search gray literature and conference abstracts. We will use broad search terms, consult a health sciences librarian, perform manual searches, and contact authors for additional data or clarification.

### Ethics and dissemination

As this study is based on a pooled analysis of previously published studies, it does not involve direct interaction with human subjects. Therefore, the ethical considerations associated with primary research, such as informed consent and privacy protection, are not directly applicable. The results of this study will be disseminated through a peer-reviewed publication, which is the gold standard for communicating scientific findings. In addition, the findings may be presented at relevant conferences to reach a wider audience and stimulate further research in this area. The results may also be shared with healthcare providers and policy-makers to inform clinical practice and health policy.

## Conclusion

This study will provide valuable insights into the effectiveness of nurse-led digital health programs for home blood pressure monitoring in patients with a history of stroke. By pooling the results of randomized controlled trials, it will offer a robust evidence base to inform clinical practice and health policy.

## Author contributions

WZ: Writing – review & editing, Writing – original draft, Visualization, Validation, Methodology, Conceptualization. ZM: Writing – review & editing, Writing – original draft, Validation, Methodology. ZF: Writing – review & editing, Writing – original draft, Validation, Methodology, Conceptualization. BL: Writing – review & editing, Writing – original draft, Validation, Methodology.

## Footnotes

^1^https://osf.io/59xqa/
